# Supplementary material for: Voltage controlled interfacial magnetism through platinum orbits
Source: Nat Commun. 2017 Jun 23;8:15848. doi: 10.1038/ncomms15848 (PMC5490004; doi:10.1038/ncomms15848)
Supplement: Supplementary Information — Supplementary Figures, Supplementary Notes and Supplementary References [file ncomms15848-s1.pdf]

Supplementary Note 1: Voltage-controlled magnetic anisotropy in an FePt|MgO system

The thickness dependence of voltage-controlled magnetic anisotropy (VCMA) in FePt|MgO systems has been characterized. Supplementary Fig. 1a shows a schematic of the sample structure. The FePt layers were made by alternate depositions of monatomic layers of Pt and Fe. Supplementary Fig. 1b shows the FePt thickness dependence of the magnetisation of the multilayer measured by a vibrating sample magnetometer. The magnetic moment linearly increases as the FePt thickness increases.

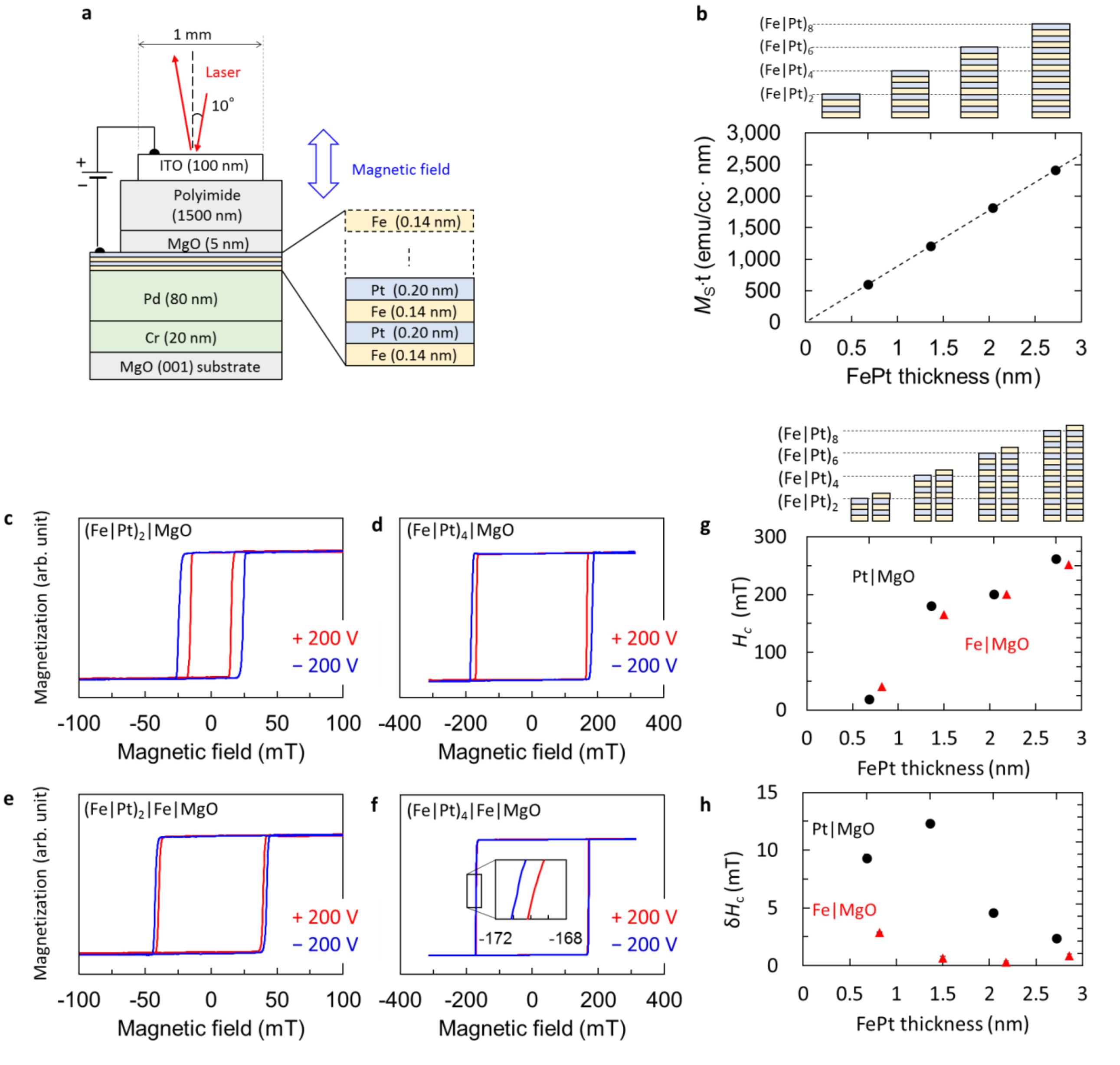

Supplementary Figure 1 | Voltage-controlled magnetic anisotropy in FePt|MgO. (**a**) A schematic of the device structure. (**b**) Magnetisation in FePt. **(c, d)** Magnetisation curves for samples with Pt-termination. (**e, f**) Magnetisation curves for samples with Fe-termination. (**g**) The thickness dependence of the coercive field (H_c). (**h**) The thickness dependence of the change in the voltage-induced coercive field (δH_c).

Supplementary Figs. 1c-1f show the magnetisation hysteresis curve as a function of the perpendicular magnetic field measured by the magneto-optical Kerr effect. Blue and red curves represent the magnetisation curves under external voltages of − 200 V and + 200 V, respectively.

All of the multilayers were perpendicularly magnetised. Magnetisation curves taken at − 200 V have larger coercive fields (H_c) than magnetisation curves taken at + 200 V. This result shows that a negative external voltage, where electrons are depleted at the FePt|MgO interface, increases the perpendicular magnetic anisotropy energy (MAE). H_c and its voltage-induced change (δH_c) are summarized in Supplementary Figs. 1g and 1h. First, H_c increases as the FePt thickness increases. This trend means that a contribution from the bulk perpendicular MAE of FePt to the total perpendicular MAE in the FePt|MgO system is larger in thicker films. In contrast, δH_c increases as the FePt thickness decreases. This result means that a contribution from the MAE at the FePt|MgO interface is dominant in a voltage-induced MAE change. The δH_c of a multilayer with Pt-termination (black circles) is larger than the δH_c of a multilayer with Fe-termination (red triangles). Experiments with $(Fe|Pt)_1$|MgO multilayers were also conducted but the $(Fe|Pt)_1$|MgO film did not show ferromagnetic behaviour.

As the aforementioned FePt|MgO systems are perpendicularly magnetised at every thickness, it is difficult to characterize the VCMA energy. As shown in Supplementary Fig. 2a, we also conducted a controlled experiment with the following epitaxial multilayer: MgO(001) substrate|MgO buffer (5 nm)|V (30 nm)|Fe (0.50 nm)|Pt (0.20 nm)|MgO barrier (2 nm)|SiO_2 barrier (5 nm)|Cr (2 nm)|Pd (5 nm). Supplementary Figure 2b shows the VCMA of the multilayer measured by XMCD at the Pt-L_3 edge under a magnetic field perpendicular to the film plane. External voltages of ± 2.6 V correspond to electric fields of ± 0.18 V nm^{-1} in the MgO dielectric. The multilayer shows a VCMA of 0.14 pJ V^{-1} m^{-1}. Hence, we could expect similar VCMA energy in $L1_0$-FePt|MgO systems. As compared with our previous study[1], the Pt|MgO interface (0.14 pJ V^{-1} m^{-1}) shows a VCMA several times larger than that of Fe|MgO (0.03 pJ V^{-1} m^{-1}).

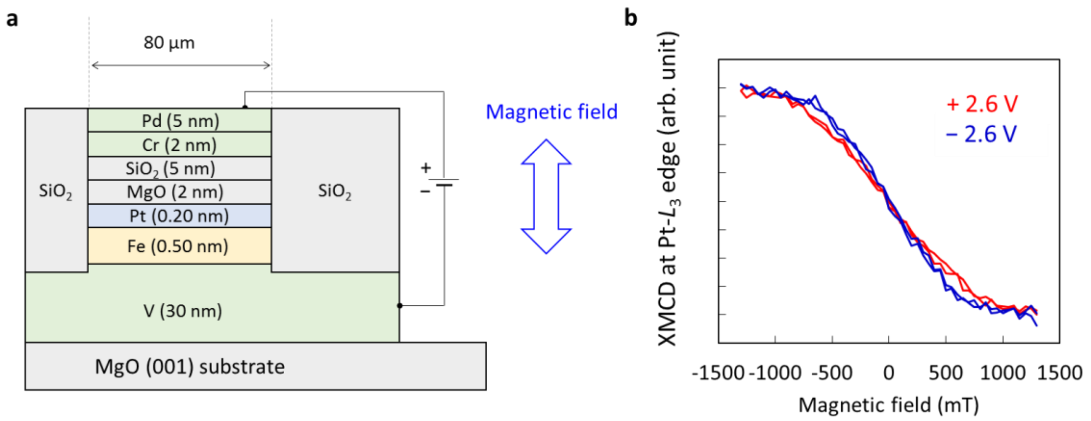

Supplementary Figure 2 | Voltage-controlled magnetic anisotropy in Fe(0.5 nm)|Pt(0.2 nm)|MgO. (**a**) A schematic of the device structure. (**b**) Magnetisation curve from the X-ray magnetic circular dichroism (XMCD) signal at the L_3 edge energy of Pt under external voltages of ± 2.6 V. Voltage-controlled magnetic anisotropy at Pt|MgO interface was 0.14 pJ V^{-1} m^{-1}.

Supplementary Note 2: STEM characterization

Supplementary Fig. 3a shows a high-angle annular dark field scanning transmission electron microscopy (HAADF-STEM) image of the FePt|MgO multilayer. The incident direction of the electron beam is in MgO [100]. We confirmed the near-total absence of lattice dislocation at the Pd|FePt interface. Then, the in-plane lattice constant of the FePt was estimated to be 0.389 nm, equal to the literature value of Pd. The in-plane lattice constant of the FePt (0.389 nm) was slightly larger than that of the literature value of 0.385 nm for $L1_0$-FePt. The FePt|MgO interface had one lattice dislocation of dozens of atoms. This dislocation is probably attributable to the 8% lattice mismatch between the literature values of $L1_0$-FePt (0.385 nm) and MgO (0.421 nm).

Supplementary Fig. 3b shows the HAADF-STEM image of FePt|MgO, where the incident electron-beam direction is in the MgO [110]. From a filtered image, we confirmed that the blight line (Mg, Pt and Pd) is connected at the FePt|MgO interface. This result shows that, in the [001] direction, the O atom in MgO is placed on the Pt atom in the FePt.

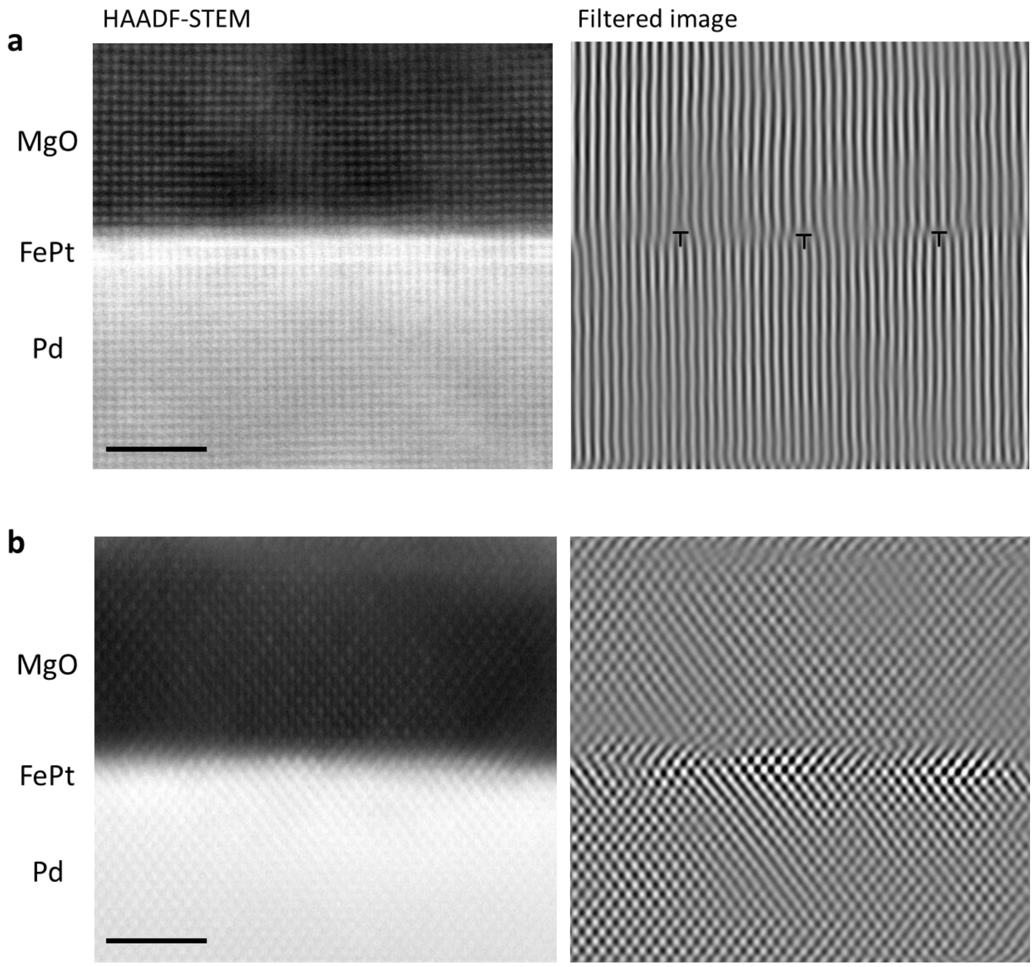

Supplementary Figure 3 | High-angle annular dark field scanning transmission electron microscopy (HAADF-STEM) image of the FePt|MgO multilayer. (**a**) The incident electron-beam direction is in MgO [100]. (**b**) The incident electron-beam direction is in MgO [110]. The scale bars indicate 2 nm.

Supplementary Note 3: Estimation of the white-line intensity of Pt XAS

The white-line intensity of Pt has been characterised from polarization-averaged XAS spectra to support quantitative characterization of the magnetic moments from the XAS/XMCD measurements (See Eqs. 1 to 3.). Because the Pt XAS spectra at the L_3 and L_3 edges show relatively small white-line peaks because of core-hole screening[2], it is difficult to characterize precisely the white-line intensity by simply subtracting arctangent functions from the measured Pt-XAS spectra. Therefore, we subtracted the XAS spectrum of Au, where the 5*d* band is nearly full, from the XAS spectrum of Pt[3]. Supplementary Figure 4 shows an example depicting both the XAS spectra of the FePt|MgO and Au bulk and their difference, where E_0 is the resonant energy at the L_3 and L_2 edges of Pt and Au. The white-line intensity (A_{L3}, A_{L2}) is defined as the integral of the Lorentzian fit of the XAS difference.

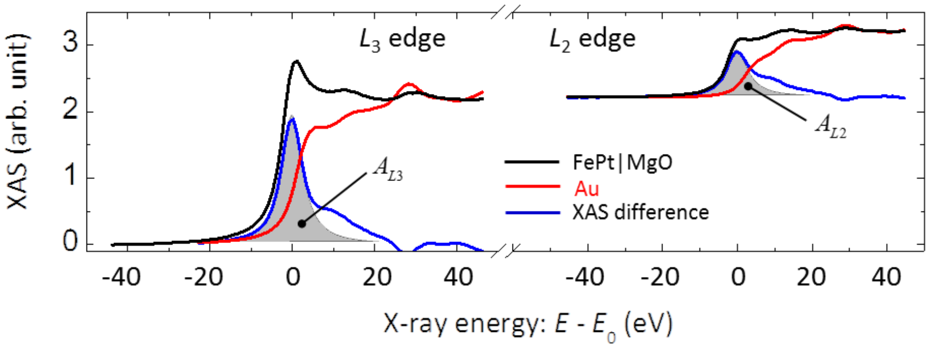

Supplementary Figure 4 | X-ray absorption spectroscopy (XAS) spectra of FePt|MgO and Au and their difference. The white-line intensity of Pt (A_{L3}, A_{L2}) can be characterized from the XAS difference.

Supplementary Note 4: Accumulated electron charge at the Pt|MgO interface

A simple capacitance model can be used to estimate either the electron charge accumulated at the Pt|MgO interface or the change in the total hole number (δn_{total}) of Pt induced by the application of a voltage. However, hard X-ray irradiation may change the dielectric constant of the polyimide, which serves as the dielectric layer in our sample. Therefore, the standard value of the dielectric constant cannot be used. We conducted a controlled experiment using a reference sample of FePt|MgO(2 nm)|SiO_2(5 nm) with a film structure similar to the target sample described in the Fig. 1b, but with a layer of MgO(2 nm)|SiO_2(5 nm) rather than MgO(5 nm)|polymide(1500 nm). The SiO_2 displays high resistance to X-ray irradiation. Thus, we assumed that the dielectric constant of MgO(2 nm)|SiO_2(5 nm) had not changed. The value of δn_{total} for the reference sample was estimated with the capacitance model[4], where the dielectric constants of 9.8 and 3.8 were employed for MgO and SiO_2, respectively.

We experimentally compared the voltage induced XAS changes at the Pt-L_3 edge of the target sample [FePt|MgO(5 nm)|polyimide(1500 nm)] with that of the reference [FePt|MgO(2 nm)|SiO_2(5 nm)]. We confirmed in the FePt|MgO|SiO_2 with an external voltage

difference of 3.9 V that the XAS difference was approximately 0.01, where the induced holes per one Pt atom was calculated from the capacitance model to be + 0.012. Figure 2b shows that the peak height of the XAS difference at the L_3 edge was approximately 0.05 in the FePt|MgO|polymide. Thus, the result of subtracting the $\delta n_{\mathrm{total}}$ of + 200 V from the $\delta n_{\mathrm{total}}$ of − 200 V in the FePt|MgO|polymide was estimated to be + 0.06. As mentioned above, we employed the dielectric constants of bulk for the calculation. As the dielectric constant of thin film depends on the film quality and is likely smaller than that of bulk, the $\delta n_{\mathrm{total}}$ might be overestimated. The error can be, at most, 10–20%.

Supplementary Note 5: Magnetic moments of Pt and Fe

Supplementary Fig. 5 shows the XAS/XMCD spectra at the Pt absorption edge of the FePt|MgO measured under perpendicular and in-plane magnetic fields. The spin magnetic moment, orbital magnetic moment and the magnetic dipole moment were characterized using sum-rule analysis. They appear in Table 1. In the analysis, we employed several relations:

$$\begin{cases} m_{\mathrm{S}}(\theta) = m_{\mathrm{S}} \\ m_{\mathrm{L}}(\theta) = m_{\mathrm{L}}^{\perp}\sin^2\theta + m_{\mathrm{L}}^{//}\cos^2\theta \\ m_{\mathrm{T}}(\theta) = m_{\mathrm{T}}^{//}(1-3\cos^2\theta) \\ m_{\mathrm{T}} \equiv m_{\mathrm{T}}^{\perp} = -2m_{\mathrm{T}}^{//} \end{cases} , \qquad (1)$$

where θ is the magnetisation angle of the FePt from the film plane. From Table 1, we obtain the result that $m_{\mathrm{L}}^{//}$ is larger than $m_{\mathrm{L}}^{\perp}$ in Pt. This observation is consistent with the fact that the orbital magnetic moment in Pt decreases the perpendicular MAE, which can be corroborated by examining $\Delta E_{\uparrow\uparrow} + \Delta E_{\downarrow\downarrow}$ in Fig. 4b.

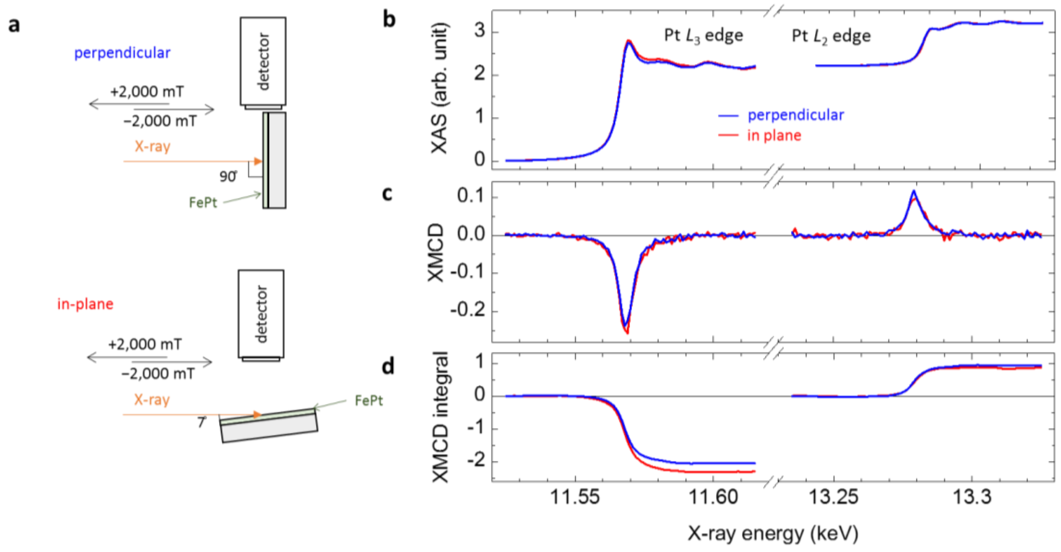

Supplementary Figure 5 | XAS/XMCD spectra at the Pt absorption edge. (**a**) Experimental setup. (**b**) XAS spectrum. (**c**) XMCD spectrum. (**d**) XMCD integrals of FePt|MgO. From these data, the spin magnetic moment, orbital magnetic moment and the magnetic dipole moment of Pt can be characterized using sum-rule analysis.

The magnetic moments of Fe in an FePt|MgO multilayer were characterized using soft XAS. To employ soft X-rays, multilayers with identical designs, except for absence of polyimide films and top electrodes, were prepared. Supplementary Fig. 6 shows the XAS/XMCD spectra at the Fe absorption edge. The spin magnetic moment, orbital magnetic moment and magnetic dipole moment of the Fe were characterized using sum-rule analysis and Supplementary Eq. 1. The results are summarized in Table 1.

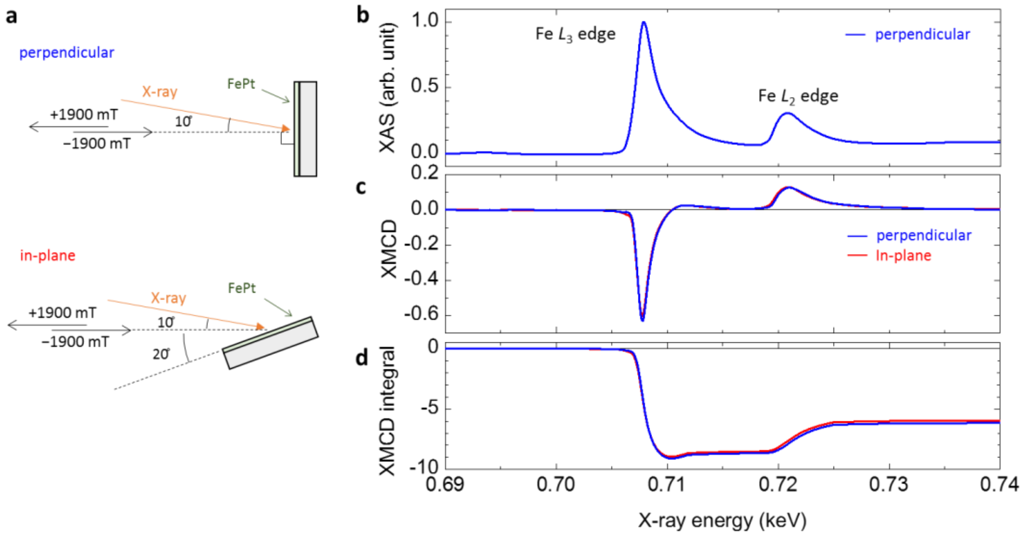

Supplementary Figure 6 | XAS/XMCD spectra at the Fe absorption edge. (**a**) Experimental setup. (**b**) XAS spectrum. (**c**) XMCD spectrum. (**d**) XMCD integrals of FePt|MgO. From these data, the spin magnetic moment, orbital magnetic moment and the magnetic dipole moment of Pt can be characterized using sum-rule analysis.

Supplementary Note 6: Magnetisation direction dependence of the voltage effect

Supplementary Fig. 7 shows the external voltage dependence of the XAS/XMCD spectra under perpendicular and in-plane magnetic fields. Supplementary Figs. 7a and 7c show the magnetisation hysteresis measured at the L_3 edge of the Pt. As shown in Supplementary Figs. 7b and 7d, similar external-voltage-induced XAS changes were observed in perpendicular and in-plane magnetised films. For the XMCD, the voltage-induced change in the perpendicularly magnetised film differs from the voltage-induced change exhibited by the in-plane magnetised film. From sum-rule analysis, the effective spin magnetic moment change, $\delta(m_S - 7m_T)/(m_S - 7m_T)$, is estimated to +13% ± 3% and −7% ± 7% in perpendicularly magnetised and in-plane magnetised films, respectively. These results indicate induction of the magnetic dipole moment in Pt by an external voltage.

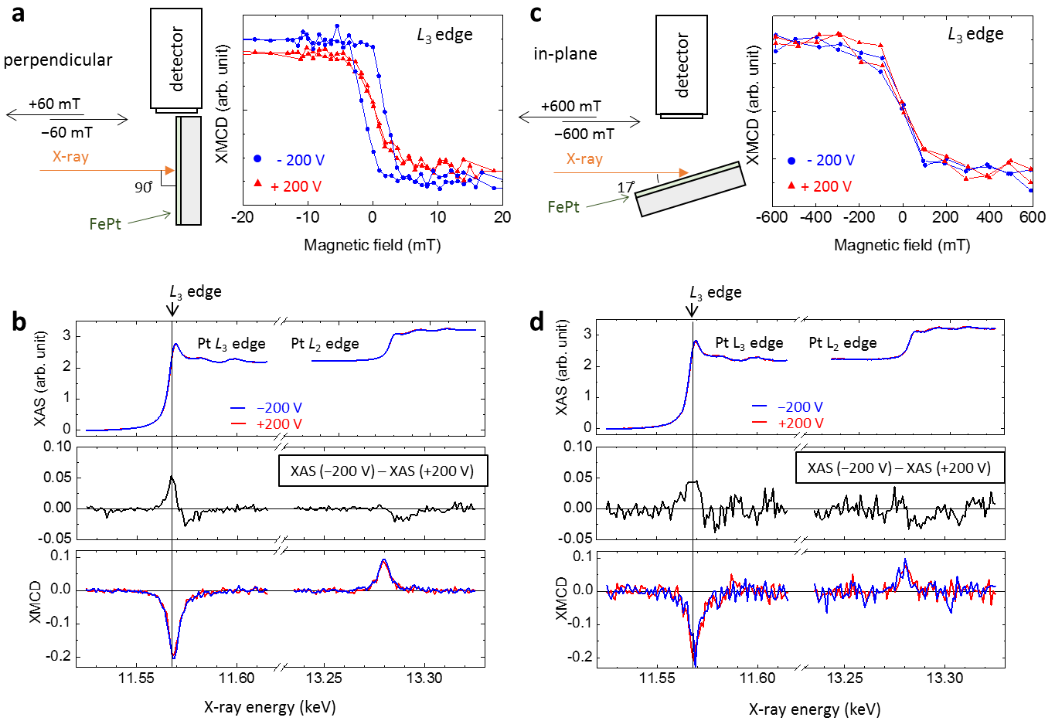

Supplementary Figure 7 | | Magnetisation direction dependence of the XAS/XMCD spectra at the Pt absorption edge under external voltages. (**a, c**) Experimental setup and element-specific magnetisation hysteresis curves of FePt|MgO measured at the Pt L_3 edge. (**b, d**) XAS, XMCD and XMCD integrals of FePt|MgO.

Supplementary Note 7: Theoretical study

The computational model for the first-principles study is depicted in Fig. 4a. Each magnetic moment in Supplementary Fig. 8 is defined as the average of Pt-1 and Pt-2. Supplementary Fig. 8a shows the perpendicular MAE of FePt, which is defined as the sum of Pt-1, Fe-1, Pt-2 and Fe-2. The $\delta n_{\mathrm{total}}$ is the induced holes of the total electron orbital of Pt-1. As in the experiment, a negative voltage (electron depletion at Pt|MgO interface) increases the perpendicular MAE. The VCMA coefficient in the system is − 0.28 pJ V^{-1} m^{-1}.

Supplementary Figs. 8b, 8c and 8d show the orbital magnetic moments of Pt. Supplementary Fig. 8d and Eq. 5 provide the result that the voltage-induced change in the orbital magnetic moment should decrease for the perpendicular MAE. This result is consistent with the result, $\delta E_{\uparrow\uparrow} + \delta E_{\downarrow\downarrow}$, displayed in Fig. 4c because the perpendicular MAE change arising from the orbital magnetic moment corresponds to the spin-conserved terms in Eq. 6. Supplementary Fig. 8e shows the voltage induction of the spin magnetic moment (m_{S}). The voltage-induced change can be seen but is independent of the magnetisation direction. Supplementary Figs. 8f and 8g show the magnetic dipole moment ($-7m_{\mathrm{T}}$) and its magnetisation direction dependence ($m_{\mathrm{T}}^{\perp} - m_{\mathrm{T}}^{//}$). The anisotropy in Supplementary Fig. 8g and Eq. 5 explains the enhanced perpendicular MAE under a negative external voltage. This explanation is consistent with the results, $\delta E_{\downarrow\uparrow} + \delta E_{\uparrow\downarrow}$, in Fig. 4c because the perpendicular MAE change by the

magnetic dipole moment is associated with the spin-flip terms in Eq. 6. Supplementary Figs. 8h and 8i show orbital magnetic moment (m_L (= $m_L^{\downarrow} + m_L^{\uparrow}$)) and effective spin magnetic moment ($m_S - 7m_T$), respectively. These are the physical parameters which can be characterized by XMCD experiments. While the voltage-induced change in the anisotropy of the orbital magnetic moment is negligible, that of the effective spin magnetic moment is distinct.

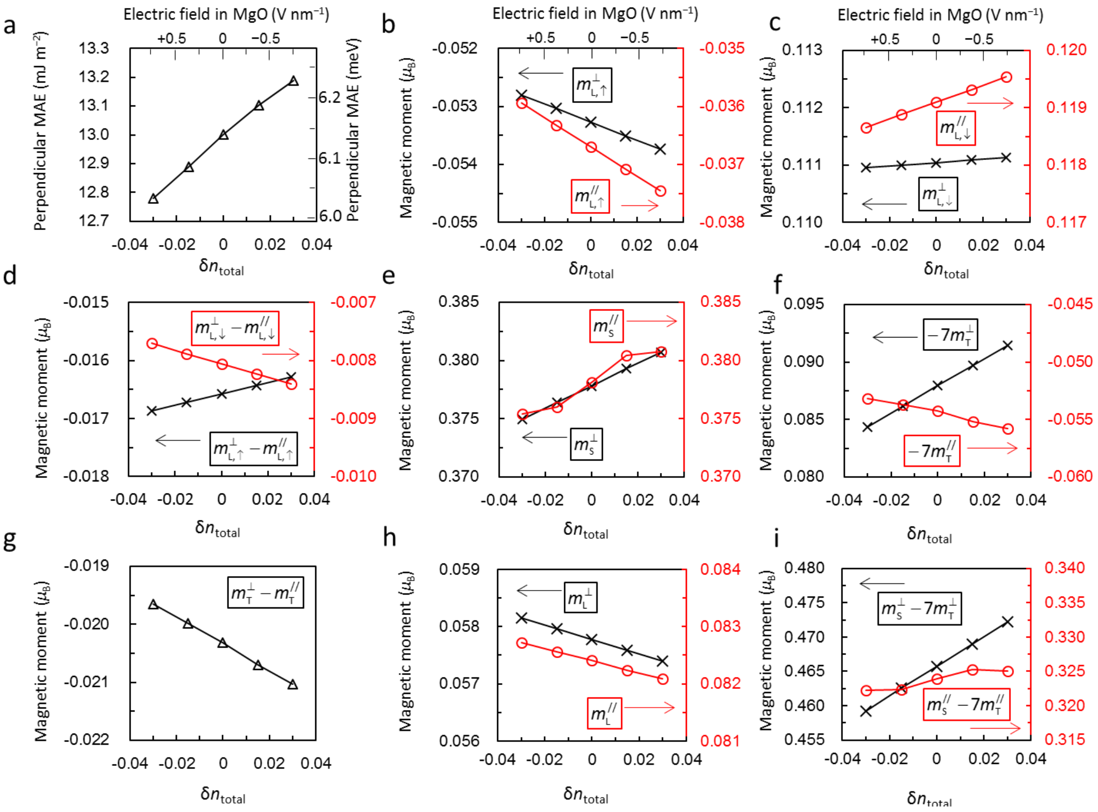

Supplementary Figure 8 | Perpendicular MAE of FePt and the magnetic moments of Pt by the first-principles study. (**a**) Perpendicular MAE of FePt as a function of the accumulated electrons at the Pt|MgO interface. (**b–d**) Orbital magnetic moment (m_L) from the majority (↑) and minority (↓) spin-bands where the magnetisation is perpendicular (┴) and in-plane (//) to the film. (**e**) Spin magnetic moment (m_S). (**f, g**) Magnetic dipole moment (m_T). (**h, i**) Orbital magnetic moment (m_L (= $m_L^{\downarrow} + m_L^{\uparrow}$)) and effective spin magnetic moment ($m_S - 7m_T$), which can be characterized by XMCD experiments.

Supplementary References

1. Miwa, S. *et al.* Voltage-controlled magnetic anisotropy in Fe|MgO tunnel junctions studied by x-ray absorption spectroscopy. *Appl. Phys. Lett.* **107**, 162404 (2015).
2. Tamura, E., van Ek, J., Fröba, M. & Wong, J. X-Ray Absorption Near Edge Structure in Metals: Relativistic Effects and Core-Hole Screening. *Phys. Rev. Lett.* **74**, 4899–4902 (1995).
3. Bartolomé, J. *et al.* Magnetisation of Pt_{13} clusters supported in a NaY zeolite: A XANES and XMCD study. *Phys. Rev. B* **80**, 014404 (2009).
4. Maruyama, T. *et al.* Large voltage-induced magnetic anisotropy change in a few atomic layers of iron. *Nat. Nanotechnol.* **4**, 158–161 (2009).
